# Selection and Identification of Novel Aptamers Specific for Clenbuterol Based on ssDNA Library Immobilized SELEX and Gold Nanoparticles Biosensor

**DOI:** 10.3390/molecules23092337

**Published:** 2018-09-13

**Authors:** Xixia Liu, Qi Lu, Sirui Chen, Fang Wang, Jianjun Hou, Zhenlin Xu, Chen Meng, Tianyuan Hu, Yaoyao Hou

**Affiliations:** 1 Hubei Key Laboratory of Edible Wild Plants Conservation and Utilization, Hubei Normal University, Cihu Road, Huangshigang District, Huangshi 435002, China; liuxixia1@163.com (X.L.); 18271691835@163.com (Q.L.); 17371958850@163.com (S.C.); WF_0129@163.com (F.W.); 13329967633@163.com (C.M.); 18771808527@sina.cn (T.H.); dxhe@163.com (Y.H.); 2 Guangdong Provincial Key Laboratory of Food Quality and Safety, South China Agricultural University, Wushan Road, Tianhe District, Guangzhou 510642, China

**Keywords:** clenbuterol, systematic evolution of ligands by exponential enrichment, real-time quantitative PCR, high-throughput sequencing technology, aptamers, gold nanoparticles biosensor

## Abstract

We describe a multiple combined strategy to discover novel aptamers specific for clenbuterol (CBL). An immobilized ssDNA library was used for the selection of specific aptamers using the systematic evolution of ligands by exponential enrichment (SELEX). Progress was monitored using real-time quantitative PCR (Q-PCR), and the enriched library was sequenced by high-throughput sequencing. Candidate aptamers were picked and preliminarily identified using a gold nanoparticles (AuNPs) biosensor. Bioactive aptamers were characterized for affinity, circular dichroism (CD), specificity and sensitivity. The Q-PCR amplification curve increased and the retention rate was about 1% at the eighth round. Use of the AuNPs biosensor and CD analyses determined that six aptamers had binding activity. Affinity analysis showed that aptamer 47 had the highest affinity (Kd = 42.17 ± 8.98 nM) with no cross reactivity to CBL analogs. Indirect competitive enzyme linked aptamer assay (IC-ELAA) based on a 5′-biotin aptamer 47 indicated the limit of detection (LOD) was 0.18 ± 0.02 ng/L (n = 3), and it was used to detect pork samples with a mean recovery of 83.33–97.03%. This is the first report of a universal strategy including library fixation, Q-PCR monitoring, high-throughput sequencing, and AuNPs biosensor identification to select aptamers specific for small molecules.

## 1. Introduction

Clenbuterol (CBL) is a beta-adrenergic receptor agonist originally used to treat asthma [1]. After CBL enters the body of livestock and poultry, it promotes the growth of animals and increases the lean meat rate [2]. However, food poisoning can result from the human consumption of the edible parts of animals containing CBL. China’s Ministry of Agriculture banned the use of adrenaline in animal production in March 1997. However, driven by economic interests, excess CBL in livestock and poultry products frequently causes human poisoning. Therefore, it is necessary to monitor the residue of CBL in livestock and poultry products over a long period. CBL residues are mainly found in livestock, and there is a high demand for domestic animal foods; thus, the detection of CBL residue-related products needs to be rapid. Currently, gold immunochromatography strips [3] and enzyme linked immunosorbent assay [4] are commonly used for this purpose. The key reagents of these two domain detection methods are antibodies. However, the production of antibodies is extremely complex and batch-to-batch variation occurs. Therefore, it is necessary to identify molecules that function similar to antibodies for the development of rapid detection products.

Aptamers are single-stranded DNA (ssDNA) or RNA selected and prepared by Systematic Evolution of Ligands by Exponential Enrichment (SELEX) [5]. Compared with antibodies, they have many advantages including different classes of target, easy modification, timesaving, strong specificity, and high affinity [6]. In the field of food detection, they have an extremely broad usage for the detection of small molecules, and they rival antibodies to some degree. Aptamers have been used to detect small molecules including patulin [7], ochratoxin [8], ractopamine [9] chloramphenicol [10], clenbuterol [11], aflatoxin B1 [12], and tetracycline [13]. The ochratoxin aptamer [14,15,16] is the most commonly used aptamer, also known as the star aptamer. Currently, there are insufficient numbers of aptamers to detect small molecules residual in food, because of their small molecular weight. How to quickly and efficiently select and identify novel aptamers specific for small molecules is an important question that needs to be answered urgently.

Many SELEX techniques are used to select aptamers, such as magnetic bead SELEX [17], graphene oxide (GO) SELEX [18], capillary electrophoresis SELEX [19], and affinity chromatography SELEX [20]. These methods mainly fix target molecules on the surface of carriers, and then enrich the specific aptamers in the library. Compared with biological macromolecules, the fixation of small molecules is difficult. Before the fixation, small molecules must be derivated, and a carboxyl group or amino active group generated. Then, they are coupled with a solid phase carrier. However, the selected aptamers often recognize the derivatives rather than the small molecules themselves, and therefore cannot be used for the selection of specific aptamers. To solve this problem, a previous study fixed the library on the surface of a solid phase carrier and small molecules were used to capture the aptamers on the solid phase carrier surface [21,22]. This method allowed the simultaneous selection of multiple targets and was also used to select aptamers against small molecules. The current method is to fix the library on the surface of magnetic beads [23] or graphene oxide (GO) [24]. Compared with GO, the magnetic bead method is based on the binding principle of streptavidin-biotin affinity. The library is more stable for fixation and elution compared with GO, and the number of selection rounds is reduced, which saves the selection cost and time. Duan [23] used self-made magnetic beads to select an aptamer specific for CBL. However, there were too many selection rounds (16 rounds). In addition, conventional cloning and sequencing were used to pick out candidate aptamers, resulting in few aptamer candidate sequences. Furthermore, fluorescence labeled aptamers and GO were used for affinity identification, although the cost of the fluorescence labeled aptamer was high. We propose that unmodified gold nanoparticles (AuNPs) based colorimetric biosensors could be used to identify active aptamers from mass enriched sequences. The principle is that AuNPs aggregate after an aptamer specifically combines with its target and the color changes from red to purple (blue). This method is simple, cheap, and quick, and it is suitable for picking out active aptamers from candidate aptamers. Although this method has been used to detect different kinds of small molecules [25,26], no report has identified active aptamers specific for small molecules from an enriched library using this method.

This study reports the first use of a multiple combination selection and identifying strategy, including ssDNA library fixation, Q-PCR monitoring, high-throughput sequencing and AuNPs biosensor identification. This universal technology allows the rapid selection and identification of novel aptamers specific for small molecules. This multiple combination selection and identifying strategy was used to select aptamers specific for CBL, and the specific aptamer successfully detected residual CBL in a pork sample.

## 2. Results

### 2.1. Magnetic Bead SELEX Selection of an Aptamer Specific for Clenbuterol

**Figure 1:**
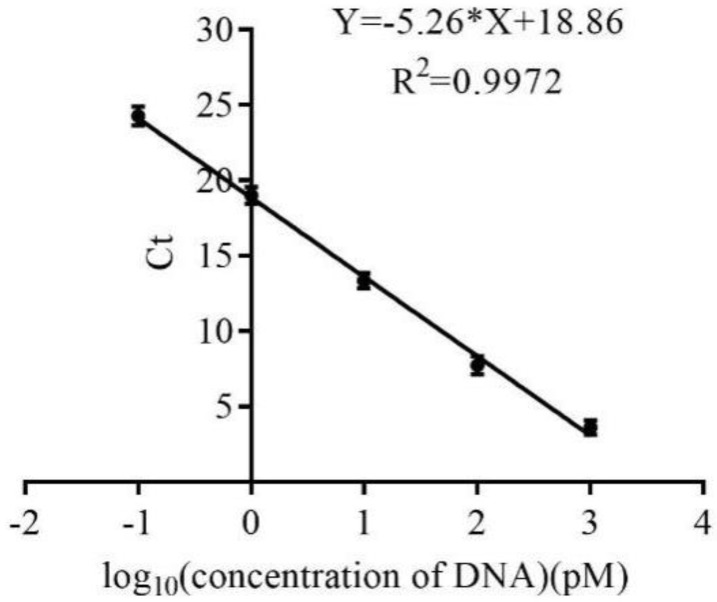
The quantitative standard curve of Q-PCR.

**Figure 2:**
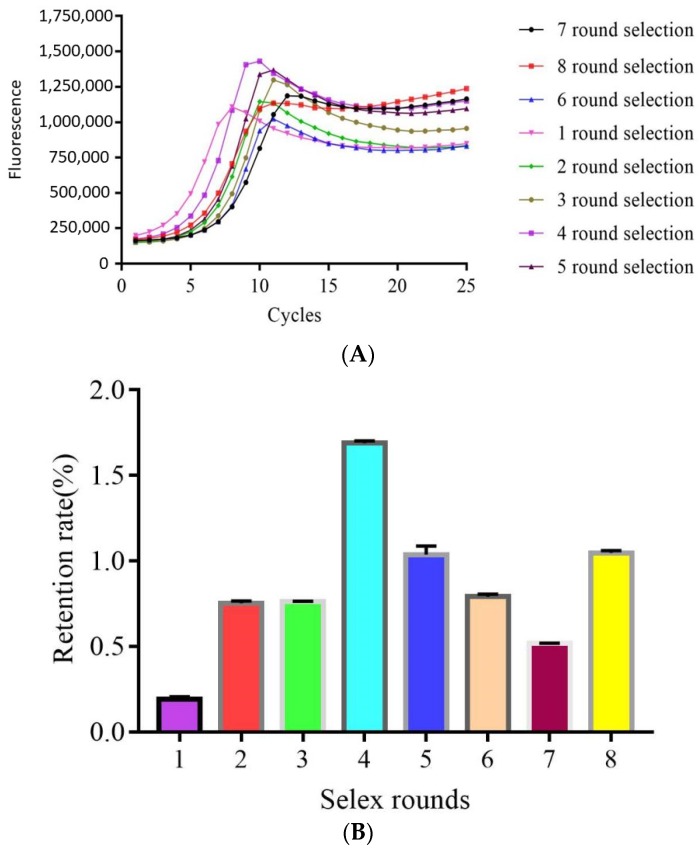
Q-PCR monitoring of the selection process: (**A**) amplification curve of Q-PCR for each round of selection; and (**B**) retention rate for each round of selection.

Q-PCR was used to monitor the selection process according to the Ct values and amplification curve for qualitative and quantitative analysis. The quantitative standard curve is shown in Figure 1 and the standard curve correlation coefficient *R*^2^ was 0.9972, showing that the linear correlation between Ct value and logarithm DNA concentration of the template by Q-PCR was good. Therefore, this method can be used for the quantitative calculation of the enriched library amount for each round. The amplification curve and retention rate for all eight rounds of SELEX selection are shown in Figure 2. Figure 2A shows that the amplification curve changed with an increase in enriched rounds. The amplification curve was used to determine when to stop selection, and was related to the initial concentration of the eluent library, the counter selection conditions and specific or non-specific binding. For these reasons, the amplification curves were disordered, but this did not prevent us determining when to stop the selection. According to a report by Luo [27], the diversity of DNA libraries decreased during the SELEX selection, and this reduced mismatching of the DNA sequences. Consequently, the drop-in fluorescence disappeared and the change in fluorescence reached a plateau. Changes in the amplification curves directly represent the convergence of the aptamer species during the SELEX procedure. Therefore, in our study, by the end of the eighth round of selection, the amplification curve showed that the fluorescence had increased, which indicated that the enriched aptamer library was specific for CBL; thus, the selection was stopped. Figure 2B shows that the retention rate increased gradually from the first to fourth round of selection because the library was enriched for specific and non-specific binders. After adding counter selection, the retention rate decreased gradually from the fifth to seventh round of selection compared with the fourth round of selection because most of the non-specific binders were selected out. For the eighth round of selection, the retention rate was increased because this enriched library contained the most specific binders although counter selection was added. In summary, the amplification curve and retention rate indicated that the aptamer library was enriched specifically after eight rounds of selection.

### 2.2. High-Throughput Sequencing and Sequence Analysis of the Enriched Library

High-throughput sequencing technology was used to sequence the enriched library of the eighth round. Overall, 105 sequences were obtained, and 172 sequences with a greater frequency of occurrence were selected, as shown in Figure S1. The 40 bp random area sequences underwent homologous alignment using clustal software, and the sequence homology rate was the highest when two sequences were nearest. Phylogenetic tree analysis of these sequences is shown in Figure S2. After removing the sequences that could not be clustered with other sequences, 104 sequences were selected. The mfold online analysis platform showed that 63 aptamers contained one ring region structure or more, and these were selected to identify binding activity. To save the cost of sequence synthesis, 11 bp were removed from the 5′ and 3′ constant regions of the aptamers. The truncated aptamers with 58 bp remaining had unchanged secondary structures, and these were used for binding activity analysis.

### 2.3. Establishment of a Gold Nanoparticles Biosensor to Identify Active Aptamers

**Figure 3:**
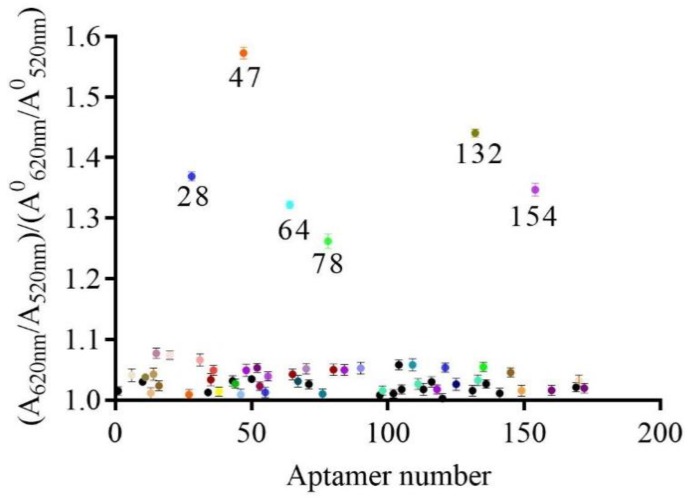
Preliminarily selection of active aptamers using the AuNPs biosensor.

The preliminarily selection results of the AuNPs biosensor are shown in Figure 3. The ratio between the experimental group and the blank group was close to 1 for most of the aptamers, indicating no binding activity between CBL and aptamers, and that the aptamer had no affinity. Interestingly, six aptamers had a ratio of >1.1 between the experimental group and the blank group. The results indicated that the A_620 nm_/A_520 nm_ value of the experimental group was greater than that of the blank group, which indicated that CBL and the aptamers produced a large number of combined complexes. Thus, these six aptamers had affinity with CBL. This method of identifying the binding activity of aptamers specific for a small molecule is the first to identify aptamers from an enriched library. The method is simple, rapid, and low-cost, and can be used to identify other active aptamers from the enriched library.

### 2.4. Characterization of Active Aptamers

**Figure 4:**
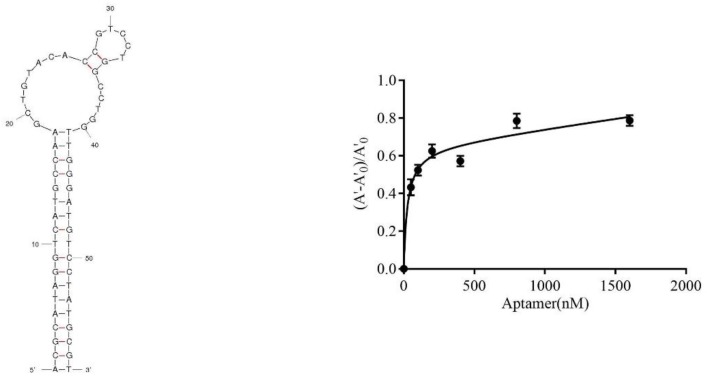
The secondary structure predicted by mfold and corresponding saturation curve of aptamer 47.

The sequence information of the six active aptamers are shown in Table 1. The secondary structures of these six aptamers were analyzed on the mfold platform, which showed that they contained two ring regions and Gibbs free energy (Table 1). The affinities of these six aptamers were analyzed, and the affinity constants were different. The affinity of aptamer 47 was the highest, with a Kd = 42.17 ± 8.98 nM. The secondary structure and affinity curve of aptamer 47 are shown in Figure 4.

**Figure 5:**
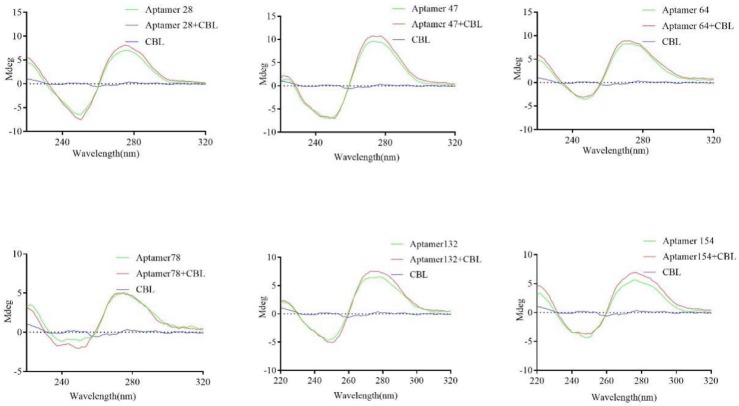
CD spectrum identification of binding activity between six aptamers and CBL.

To confirm the binding activity between CBL and aptamers, conformation changes of the aptamer before and after CBL binding were studied by circular dichroism (CD) spectrum, a powerful and sensitive tool for studying the interaction between DNA and small molecules [28]. Figure 5 shows that CBL has an extremely weak CD signal, while the aptamer presents a positive and negative peak at about 275 nm and 250 nm, respectively, which was consistent with the reported CD spectrum of a single chain oligonucleotide [29]. When the ssDNA-CBL complex was formed by the binding of free CBL with the aptamer, the characteristic peak at 275 nm was significantly increased, while the characteristic peak at 250 nm was only slightly changed. This suggests that the binding of CBL to the aptamer embedded form led to the reduction of DNA base accumulation. Therefore, the aptamer conformation changed after binding with CBL, which confirmed the interaction between the six aptamers and CBL.

**Figure 6:**
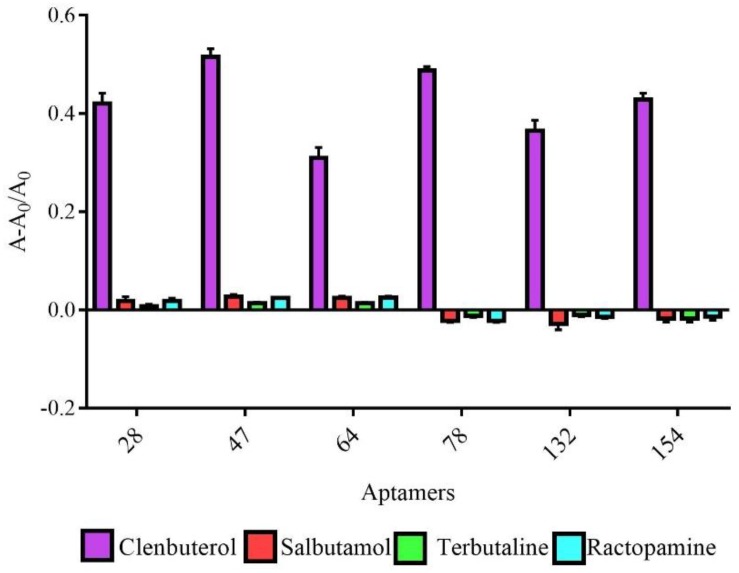
Identification of the specificity of six active aptamers against CBL.

The specific analysis results are shown in Figure 6. The response signals of the six aptamers binding with CBL exceeded that observed with the CBL analogs, which indicated that there was no cross reaction with the CBL analogs and that the six aptamers had high specificity.

### 2.5. Detection of CBL Using a Gold Nanoparticles Biosensor and Indirect Competitive Enzyme Linked Aptamer Assay

**Figure 7:**
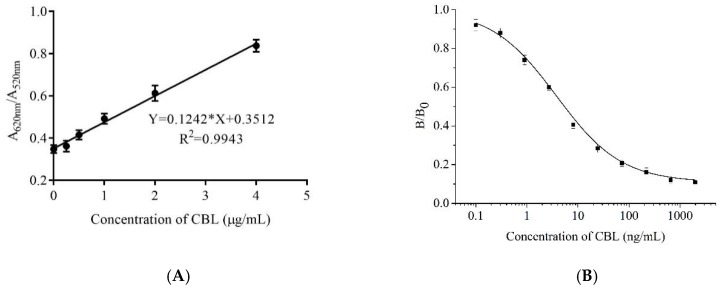
Standard curve for CBL detection: (**A**) standard curve for CBL detection with aptamer 47 using AuNPs biosensor; and (**B**) standard curve for CBL detection with 5′-biotin aptamer 47 using ic-ELAA.

The sensitivity analysis of aptamer 47 and biotin-aptamer 47 are shown in Figure 7. The AuNPs biosensor results are shown in Figure 7A. The limit of detection (LOD) was 1.2 ± 0.005 ng/mL (n = 3), as estimated by the equation LOD = 3SD/slope, where SD represents the standard deviation of the blank, and the slope was obtained from the calibration curve. The linear detection range was 0.01–4 μg/mL. However, this method was affected by the salt concentration, and therefore was not suitable for the detection of real samples. The complex sample matrix in food may cause AuNPs to change color; therefore, it is necessary to establish another method for real sample detection.

The results of the indirect competitive enzyme-linked aptamer assay (ic-ELAA) are shown in Figure 7B. The mean concentration of CBL required for 50% inhibition of binding (IC_50_) and LOD (LOD for 10% inhibition of binding) were 5.44 ± 0.01 ng/mL (n = 3) and 0.18 ± 0.02 ng/L (n = 3), respectively, and the linear response range extended from 0.61 to 92.20 ng/mL, showing that the 5′-biotin aptamer 47 could be used to develop ic-ELAA. We used the 5′-biotin aptamer 47 for pork analysis (Table 2). The mean recovery was 83.33–97.03%, indicating that the 5′-biotin aptamer 47 was suitable for the detection of residual CBL in real samples.

## 3. Discussion

SELEX selection generally has to undergo multiple rounds of library enrichment. It is very important to monitor whether the specific aptamer is enriched in the selection process. At present, the most widely used method is Q-PCR, and studies by Mencin [30] and Michiels [31] monitored the SELEX progress according to the melt curve of Q-PCR. However, Luo [27] found that a single melting curve analysis was insufficient to monitor a pool especially during small molecule aptamer selection, because the melting curve did not change during small molecule aptamer selection. They monitored the selection process using the Ct value and Q-PCR amplification curve. When the library presented with specific enrichment, the Ct value was significantly reduced and the amplification curve increased. This method was suitable to monitor the selection progress of aptamers specific for different classes of targets. However, it has not been used to monitor the selection of aptamers specific for CBL. In our study, we used this method to monitor SELEX progress, and after only eight rounds of selection, SELEX was stopped. The number of selection rounds was lower than reported by a previous report for small molecule selection, which used 10–16 rounds. Therefore, this method might save time and cost, and aptamer selection was simple.

The preparation of a secondary ssDNA library is a key step of SELEX technology, and existing methods include asymmetric PCR [32], lambda exonuclease digestion [33], and unequal length primer PCR [34]. Among them, the asymmetric PCR method is often accompanied by the generation of double-stranded DNA during the preparation of ssDNA. The purity of the secondary library is not high and it is difficult to accurately determine concentrations. The lambda DNA enzyme method has high efficiency, but the enzyme cutting system is large, the cost of enzymes is high, and thus the selection cost is greatly increased. In the current study, we used unequal length primer PCR combined with fluorescence labeling technology to add a fluorescence label to short primers. Through PAGE degenerative electrophoresis separation, fluorescence bands were cut and prepared as for a secondary ssDNA library. This allows the secondary ssDNA library to be separated rapidly and at low cost. The main disadvantage of conventional PCR amplification during unequal length primer PCR is that overamplification increases nonspecific hybridization among different products and by-products, which may cause the loss of potential specific aptamers, inefficient selection, and even selection failure. It was reported [35] that emulsion PCR could overcome the shortcomings of conventional PCR. During emulsion PCR, different templates are separated by emulsion particles, allowing single-molecule PCR, and avoiding nonspecific hybridization. In our study, emulsion PCR was used for the preparation of a secondary ssDNA library.

After multiple rounds of SELEX selection, the active candidate aptamers were picked out. Currently, the conventional method for this is T cloning [36], which randomly picks a certain number of clones for sequencing and activity identification. The disadvantages of this method are that a limited number of clones were picked and some clones were duplicated. Therefore, in our study, high-throughput sequencing technology was used to analyze sequence information in the enriched library containing a large amount of sequence information, to avoid the multiple identification of repeated clones.

A method to identify the binding active aptamers from the enriched library is critical for aptamer selection. The AuNPs biosensor based on aptamers had the advantages of being simple, rapid, sensitive, and visible, which was convenient for the development of portable and on-site rapid detection [37,38]. Of note, the free-labeled aptamers could be identified, which saved cost and time. Therefore, this method was used to batch identify aptamers from the enriched library in our study. By using this convenient method, six novel aptamers were discovered, and aptamer 47 had the highest affinity. For the AuNPs biosensor based on aptamer 47, the LOD was lower than that of Han’s patent [39] with a LOD = 4 μg/mL, and which used the ELAA method. Therefore, aptamer 47 has a greater potential for the development of a rapid detection method than the aptamer reported by Han. However, the AuNPs biosensor is not suitable for real sample detection. First, the sample matrix causes AuNPs to aggregate and change color, although this can be prevented by blocking the nanoparticle surface including adding DNA Spacer and changing aptamer graft density on AuNPs surface [40] to ensure greater stability in the conjugated colloids. However, this modification process is complicated. Second, the LOD of the AuNPs biosensor in our study was high and could not be used to detect samples with a low concentration of CBL residues. To solve this issue, we established an ic-ELAA based on the 5′-biotin aptamer 47. In the ic-ELAA analysis, the LOD was slightly higher than a previously reported aptamer with a LOD = 0.07 ng/mL [11] for CBL detection. The sequence of aptamer 47 is novel and the affinity constant is lower than a previously reported aptamer. The 5′-biotin aptamer 47 was also suitable for the development of a rapid detection kit to monitor low levels of residual CBL in pork samples. The sensitivity of the ic-ELAA was higher compared with the AuNPs biosensor, possibly because the reaction system and analysis principles were different. To avoid the interference of salt when using the AuNPs biosensor, aptamer 47 was reacted with CBL in water. For the ic-ELAA, the 5′-biotin aptamer 47 was reacted with CBL in binding buffer. Therefore, the sensitive ic-ELAA method is suitable for real sample detection.

## 4. Materials and Methods

### 4.1. Chemicals and Reagents

The clenbuterol and ractopamine standards were purchased from Shanghai Yuanye Biotechnology Ltd. (Shanghai, China). Terbutaline and salbutamol standards were purchased from Aladdin Co., Ltd. (Shanghai, China). Taq polymerase, dNTPs and 2× TBE-urea buffer were purchased from Sangon Biotech Ltd. (Shanghai, China). Streptavidin labeled magnetic beads were purchased from Thermo Fisher Scientific Ltd. (Shanghai, China). HAuCl_4_·4H_2_O was purchased from Sinopharm Chemical Reagent Co., Ltd. (Shanghai, China). Evagreen was purchased from Shanghai Open Biotechnology Ltd. (Shanghai, China). All other analytical pure chemical reagents were purchased from Sinopharm Chemical Reagents Ltd. (Shanghai, China). streptavidin-horseradish peroxidase was purchased from Boster Biological Technology Co. Itd. (Wuhan, China). Other solutions used in the experiments were treated with sterilization ultrapure water. The aptamer library was synthesized by Sangon Biotech Ltd. (Shanghai, China). The primers (Biotin-P, FAM-Forward, polyA-Reverse, Q-Forward, Q-Reverse, Table 3) were synthesized by Nanjing Genscript Biotechnology Ltd. (Nanjing, China). The aptamers and 5′-biotin aptamer 47 were synthesized by Suzhou Hongxun Biotechnology Ltd. (Suzhou, China). High-throughput sequencing was performed by Anhui Angputuomai Biotechnology Ltd. (Hefei, China). Secondary structures were calculated with mfold online bioinformatics platforms (http://unafold.rna.albany.edu/?q=mfold/DNA-Folding-Form).

### 4.2. Selection of Aptamers Specific for CBL Based on ssDNA Library Immobilized SELEX

**Figure 8:**
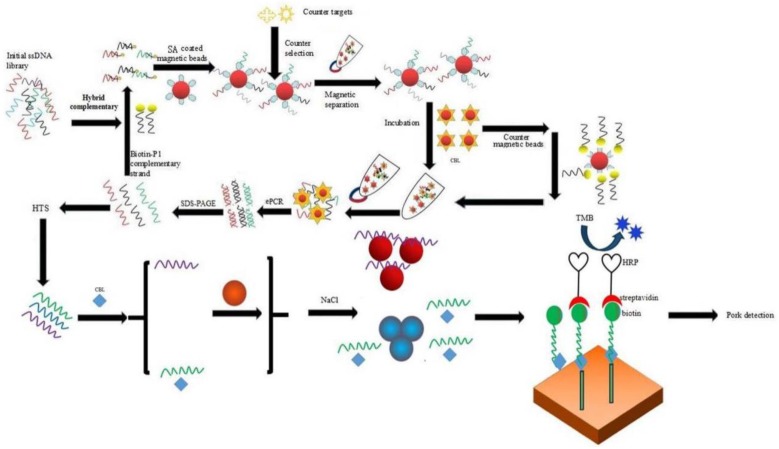
Graphical abstract.

The procedure of ssDNA Library Immobilized SELEX for aptamers against CBL is shown in Figure 8. The synthetic ssDNA library (5**′**-ATTGGCACTCCACGCATAGG(N)_40_CCTATGCGTGCTACCGTGAA-3**′**) was dissolved in binding buffer (0.1 g CaCl_2_, 0.2 g KCl, 0.2 g KH_2_PO_4_, 0.1 g MgCl_2_.6H_2_O, 8 g NaCl, and 1.15 g Na_2_HPO_4_, 1 L). Biotin-P was mixed with the library at a ratio of 2:1, and slowly denatured and renatured (95 °C for 10 min, 60 °C for 1 min, 25 °C for 1 min). The above mixture was added to the streptavidin magnetic beads and incubated (the magnetic beads were washed 4 times with the binding buffer before use, and 700 μL magnetic beads was used in the first round of selection) for 45 min. The magnetic beads were washed 6 times, and incubated for 90 min with CBL in binding buffer (200 μL) to a final concentration of 100 μM at room temperature. The supernatant was collected by magnetic separation, the magnetic beads were washed with 200 μL binding buffer containing the CBL at a final concentration of 100 μM, and the supernatant was collected using magnetic separation. The supernatant from two elutions were pooled as the eluent library with a volume of 400 μL.

### 4.3. Establishment of a Real-Time Quantitative PCR Method for the Monitoring Selection Process

A standard curve was prepared as follows: the initial ssDNA library was diluted to a concentration of 16,000, 1600, 160, 16, and 1.6 pM as a real-time quantitative PCR template. Then, 2 μL of template was mixed with 30 μL Q-PCR mix (1 μL Q-Forward at a concentration of 10 μM, 1 μL Q-Reverse at a concentration of 10 μM, 0.5 μL dNTP mix at a concentration of 10 mM, 3 μL 10× PCR buffer, 1 μL Taq DNA polymerase, 1 μL Evagreen, 15.5 μL sterile water, total volume of 30 μL), and the following Q-PCR program (StepOnePlus, ABI, Carlsbad, CA, USA) was used: 95 °C for 2 min, 95 °C for 0.5 min, 60 min at 0.5 °C, and 72 °C for 0.5 min with 25 cycles. The enriched libraries from each round were monitored using Q-PCR amplification according to the above method, and the template was the eluent library with a volume of 2 μL.

### 4.4. Preparation of Secondary Libraries

After each round of selection, all of the eluent library was added into the emulsion PCR mix (398 μL template DNA, 10 μL FAM-Forward at a concentration of 100 μM, 10 μL polyA-Reverse at a concentration of 100 μM, 40 μL dNTP mix at a concentration of 10 mM, 200 μL 10× PCR buffer, 8 μL Taq DNA polymerase, 1732 μL sterile water, total volume of 2 mL). After adding 8 mL emulsifier (1 mL EM 90, 25 μL triton X-100, 49 mL mineral oil), the emulsion was prepared by vortexing for 2 min, and left to stand for 5 min. The PCR program was as follows: (95 °C 2 min, 95 °C 1 min, 60 °C 1 min, 72 °C 1 min, 25 cycles). The PCR product was concentrated with n-butyl alcohol, then it was mixed with 2× TBE-urea buffer and boiled for 10 min. The sample was separated by denatured SDS-PAGE (400 V, 15 min). The fluorescent strip was cut off and boiled to separate the secondary ssDNA library, which was dialyzed overnight in binding buffer. After measuring of the secondary library concentration, the next round of selection was conducted. The first, second, third and fourth rounds of the selection procedures were consistent. The fifth, sixth and seventh rounds of selection were counter selection with analogs (salbutamol, ractopamine and terbutaline) and positive selection with CBL. The eighth round of selection was positive selection with CBL, and then biotin-P magnetic beads were added for counter selection. The amount of ssDNA library in the first round of selection was 1.3 nm, and in the later rounds it was 100 pM.

### 4.5. High-Throughput Sequencing and Sequence Analysis of the Enrichment Library

In the eighth round, the library was enriched and sent to Anhui Angputuomai Biotechnology Co., Ltd. for high-throughput sequencing by an Illumina high-throughput sequencing platform (HiSeq/MiSeq). Homologous comparison and evolutionary tree analysis were carried out by clustalX2 software (downloaded from http://www.clustal.org/download/current/) and then visualized using TreeView tool version 1.6.6 for 172 sequences, which were selected from high-throughput sequencing results according to the frequency of occurrence. Then, 104 sequences with a high homology rate were selected for secondary structure prediction using mfold online bioinformatics platforms (http://unafold.rna.albany.edu/?q=mfold/DNA-Folding-Form). Sixty-three sequences with several ring regions ≥2 were selected, and 11 bases were removed from the 5′ and 3′ constant regions, respectively. Each aptamer contained 58 bp and was synthesized.

### 4.6. Determination of Binding Activity between Aptamers and CBL Using a Gold Nanoparticles Biosensor

The binding activity of candidate aptamers were preliminarily determined using an AuNPs biosensor (Figure 8). AuNPs were prepared as previous report [41] by reducing chloroauric acid with sodium citrate. This was centrifuged at 12,000 r/min, 4 °C for 25 min, the supernatant was discarded, and the precipitate was dissolved in ultrapure water, as the concentration of AuNPs in ultrapure water was determined to be 8.76 nM using UV-vis spectroscopy according to the previously reported method [42]. First, 50 µL/well of aptamers were diluted with ultrapure water until the final concentration was 0.4 μM, and then they were incubated with 1 μg/mL CBL (50 µL/well) for 30 min at room temperature. AuNPs (50 µL/well, 8.76 nM) were added and incubated for 30 min at room temperature, and then 0.9 M NaCl (10 µL/well) was added. The absorbance values at 620 nm (A_620 nm_) and 520 nm (A_520 nm_) were measured on an automatic microplate reader (I3X, Molecular Devices, San Jose, CA, USA), and aptamers with binding activity were determined by the ratio of A_620 nm_/A_520 nm_ between the experimental group and the blank group.

### 4.7. Affinity Analysis of Active Aptamers

Aptamers with different concentrations (0, 50, 100, 200, 400, 800 and 1600 nM) were incubated with 1 μg/mL CBL for 30 min at room temperature. AuNPs were added and incubated for 30 min at room temperature, and then 0.9 M NaCl was added. The A_520nm_ was measured on an automatic microplate reader, (A’‒A’_0_)/A’_0_ was used as the ordinate and the aptamer concentration was used as the abscissa. The affinity constant of aptamers was calculated by Graphpad Prism 7.0 software (GraphPad Software, La Jolla, CA, USA). A’ represents the A_520nm_ value at the concentration of each aptamer and A’_0_ represents the value of A_520nm_ when the concentration of the aptamer is zero.

### 4.8. Circular Dichroism Spectrum Analysis of Active Aptamers

The aptamers were diluted to 1 μM with binding buffer, and incubated with 10 μM CBL for 30 min at room temperature. The circular dichroism of the mixture was scanned at a scanning wavelength of 220–320 nm. At the same time, the CD spectrum (J-810, Jasco, Tokyo, Japan) analysis of CBL or aptamers were performed under the same conditions.

### 4.9. Specificity Analysis of Active Aptamers

Clenbuterol, terbutaline, salbutamol, and ractopamine (1 μg/mL) were incubated with 0.4 μM of aptamers for 30 min at room temperature. AuNPs were added and incubated for 30 min at room temperature, and then 0.9 M NaCl was added. The A_620 nm_ and A_520 nm_ were measured on an automatic microplate reader. A: represents the value of (A_620 nm_/A_520 nm_) when the concentration of Clenbuterol, terbutaline, salbutamol, and ractopamine is 1 μg/mL and A_0_: represents the value of (A_620nm_/A_520nm_) when the concentration of clenbuterol, terbutaline, salbutamol, and ractopamine is 0 μg/mL.

### 4.10. Detection of CBL Using a Gold Nanoparticles Biosensor and ic-ELAA

Aptamer 47 was diluted with ultrapure water until the final concentration was 0.4 μM, and it was then incubated with a series of CBL concentrations (0.01, 0.25, 0.50, 1.0, 2.0, and 4.0 μg/mL) for 30 min at room temperature. AuNPs were added and incubated for 30 min at room temperature, and then 0.9 M NaCl was added. The A_620 nm_ and A_520 nm_ was measured on an automatic microplate reader. (A_620 nm_/A_520 nm_) was used as the ordinate and the CBL concentration was used as the abscissa.

The sensitivity of the 5′-biotin aptamer 47 was also determined by ic-ELAA (Figure 8). A microtiter plate was coated with 5 μg/mL CBL-Bovine serum albumin (BSA) in coating buffer (15 mM Na_2_CO_3_ and 35 mM NaHCO_3_, pH 9.6) at 37 °C overnight. The CBL-BSA was prepared as previously reported [43]. The plate was washed twice with washing buffer (6 g Na_2_HPO_4_·12H_2_O, 16 g NaCl, 1.2 mL Tween 20, pH 7.4, 2 L) and then blocked with 150 μL 3% (*w*/*v*) skim milk powder in PBS for 2 h. Subsequently, a series concentration of CBL standard (0, 0.1, 0.3, 0.9, 2.7, 8.1, 24.3, 72.9, 218.7, 656.1, and 1968.3 ng/mL, 50 μL) and 5′-biotin aptamer 47 (1 μM, 50 μL) were respectively added into each well, and the resulting solution was incubated for 1 h at 37 °C. After washing six times as described above, 100 μL of streptavidin-horseradish peroxidase (HRP) diluted 1:5000 was added, and the plate was incubated at 37 °C for 1 h. After washing the plate six times, 3,3′,5,5′-tetramethylbenzidine (TMB) was added to the reaction for 10 min. The reaction was stopped with 10% H_2_SO_4_, and A_450nm_ was measured on an automatic microplate reader.

The detection of CBL in real samples was also studied using ic-ELAA. Pork was purchased from a supermarket and pretreated according the following method. One hundred grams of pork was homogenized for 10 min. Next, 10 μL of CLB standard solutions at different concentrations (1, 5, and 10 ng/μL) were individually mixed with 10 g of pork paste and homogenized for 10 min. Then, 1 g of the above homogenized sample was mixed with 1 mL of 0.01 M HCl, vortexed for 2 min and centrifuged at 12,000 r/min for 10 min. The supernatant was adjusted to pH 7.4 and centrifuged at 12,000 r/min for 10 min. The supernatant was filtered through a 0.45 μm filtration membrane and collected for the next assay.

## 5. Conclusions

Aptamers are molecules that complement the function of antibodies for the development of rapid detection kits, where the biggest bottleneck is the selection of novel specific aptamers. For small molecules, it is difficult to select aptamers with specific binding activity. This is the first study to report the use of multiple combination selection and identification strategy including library fixation, Q-PCR monitoring, high-throughput sequencing, and AuNPs biosensor identification for the selection of novel specific aptamers. The selection, monitoring, and identification process was simple, intuitive, reliable, and high-throughput. After eight rounds of selection, six novel aptamers were discovered from the ssDNA aptamer library. Aptamer 47 had the highest affinity with a binding constant of 42.17 ± 8.98 nM. The ic-ELAA based on 5′-biotin aptamer 47 was used for pork sample detection with a mean recovery of 83.33–97.03%. Most importantly, the multiple combination selection and identification strategy proposed in this study is a universal technical method, which can be used for the selection and identification of other novel aptamers specific for small molecules.

## 6. Patents

Liu, X.X.; Hou, J.J.; Lu, Q.; Wang, F.; Hou, Y.Y.; Meng, C.; Hu, T.Y.; Chen, S.Y. An aptamer used to detect CBL of clenbuterol and its screening method and application. Patent 2018, Application number: 201810874140.X.

## Figures and Tables

**Table 1:** Sequence (5′−3′), dissociation constant (Kd) and dG values of aptamer candidates.

No.	Sequences	Kd (nM)	dG
28	5′ACGCATAGGATGCAACAACGCGAATGCCACCAATTCGTGCTTGTCGTGTCCTATGCGT3′	321 ± 104.5	−15.09
47	5′ACGCATAGGTCATGCCAAGCTGTACACCGTCCTGGCCTGGTTGGGATGTCCTATGCGT3′	42.17 ± 8.98	−13.76
64	5′ACGCATAGGTACACCACATCGGATCGTAATGCGTATGTGACTCAGTGTGCCTATGCGT3′	123.3 ± 14.28	−16.26
78	5′ACGCATAGGGTACGCCACAGATCGACCTCTGGTTGGTCCTGTTGTGTGGCCTATGCGT3′	201.1 ± 42.42	−16.00
132	5′ACGCATAGGGACCATGCGCAGTGAACTTGGTTCTTTGTGCTCATGTGTGCCTATGCGT3′	221.6 ± 41.87	−14.77
154	5′ACGCATAGGGCATCACACATGTCCTTGCCATTGCTGACTTGTTTGGTGTCCTATGCGT3′	268.8 ± 40.43	−15.40

**Table 2:** Recovery test results with pork samples (n = 3).

Samples	Spiked Concentration (ng/g)	Detection Concentration (ng/g)	Recovery (%)	RSD (%)
Pork	1	0.83 ± 0.02	83.33 ± 1.53	1.83
	5	4.66 ± 0.09	93.27 ± 1.70	1.82
	10	9.70 ± 0.05	97.03 ± 0.45	0.46

**Table 3:** Primer sequences.

Name	Sequences (5′-3′)
Biotin-P	5**′**-CCTATGCGTGGAGTGCCAAT-3**′**-biotin
FAM-Forward	6-FAM-5**′**-ATTGGCACTCCACGCATAGG-3**′**
polyA-Reverse	5**′**-AAAAAAAAAAAAAAAAAAAAspacer18TTCACGGTAGCACGCATAGG-3**′**
Q-Forward	5**′**-ATTGGCACTCCACGCATAGG-3**′**
Q-Reverse	5**′**-TTCACGGTAGCACGCATAGG-3**′**

## Supplementary Materials

Figure S1: The 172 sequences with a greater frequency of occurrence from high-throughput sequencing sequences. Figure S2: Phylogenetic tree analysis of 172 sequences.
