# Supplementary figures and images for: Selection and Identification of Novel Aptamers Specific for Clenbuterol Based on ssDNA Library Immobilized SELEX and Gold Nanoparticles Biosensor
Source: Molecules. 2018 Sep 13;23(9):2337. doi: 10.3390/molecules23092337 (PMC6225122; doi:10.3390/molecules23092337)

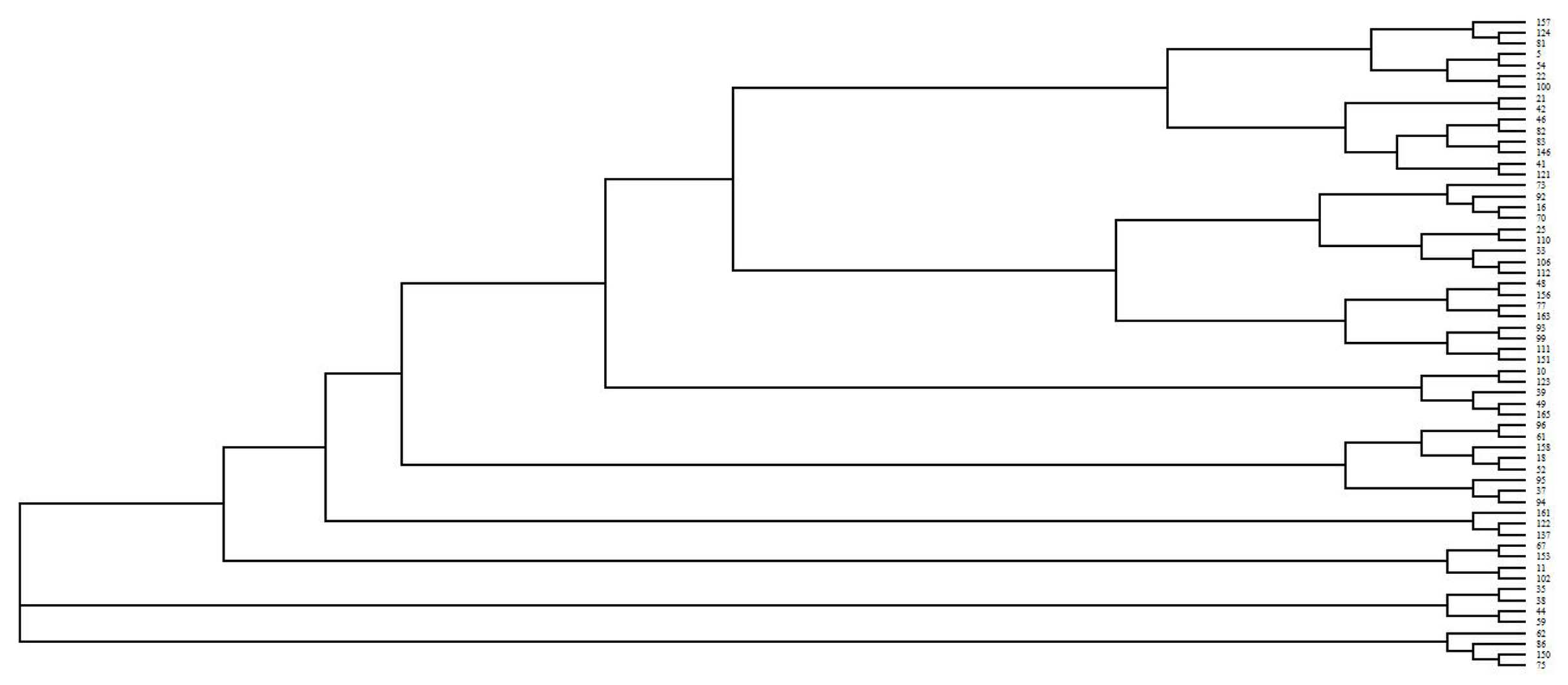

Supplement: Supplementary file 1 [file molecules-23-02337-s001.zip › Figure S2B.jpg]

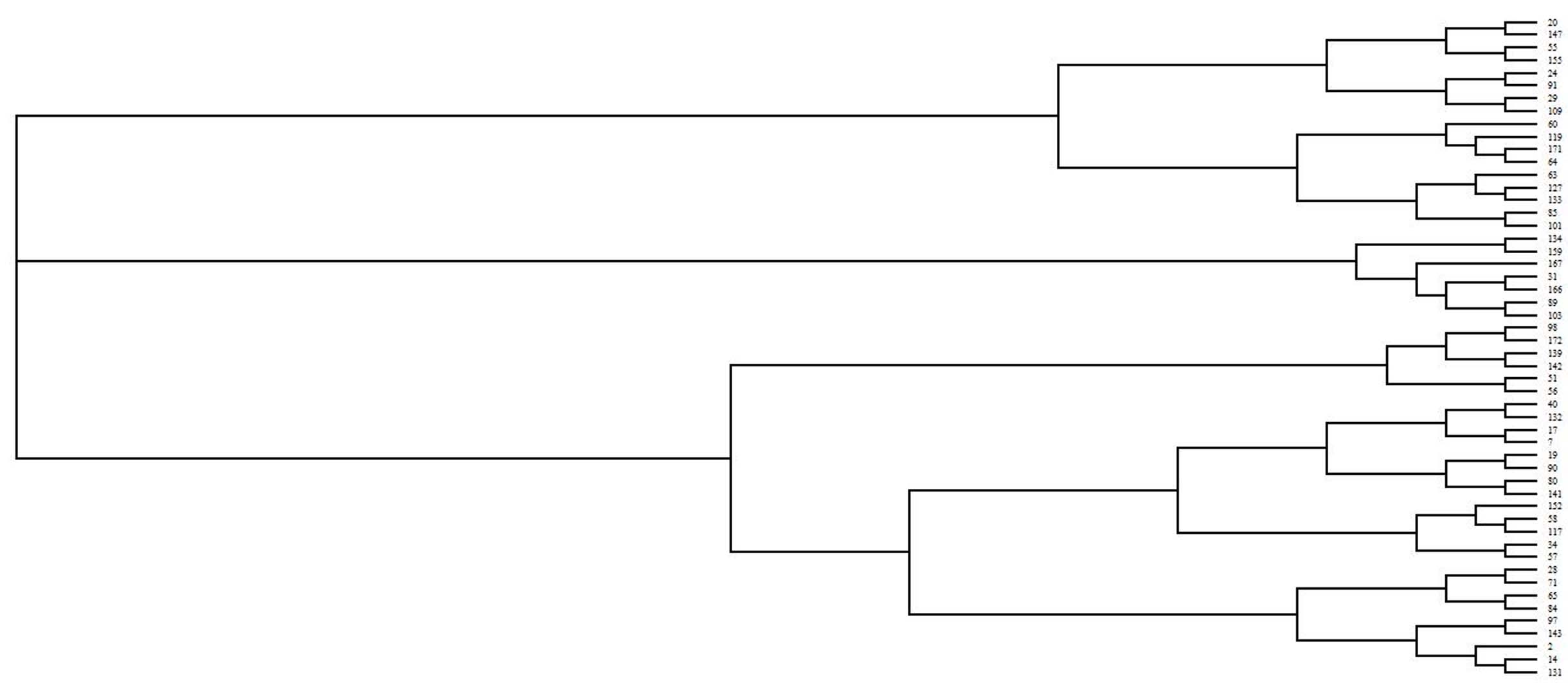

Supplement: Supplementary file 1 [file molecules-23-02337-s001.zip › Figure S2C.jpg]

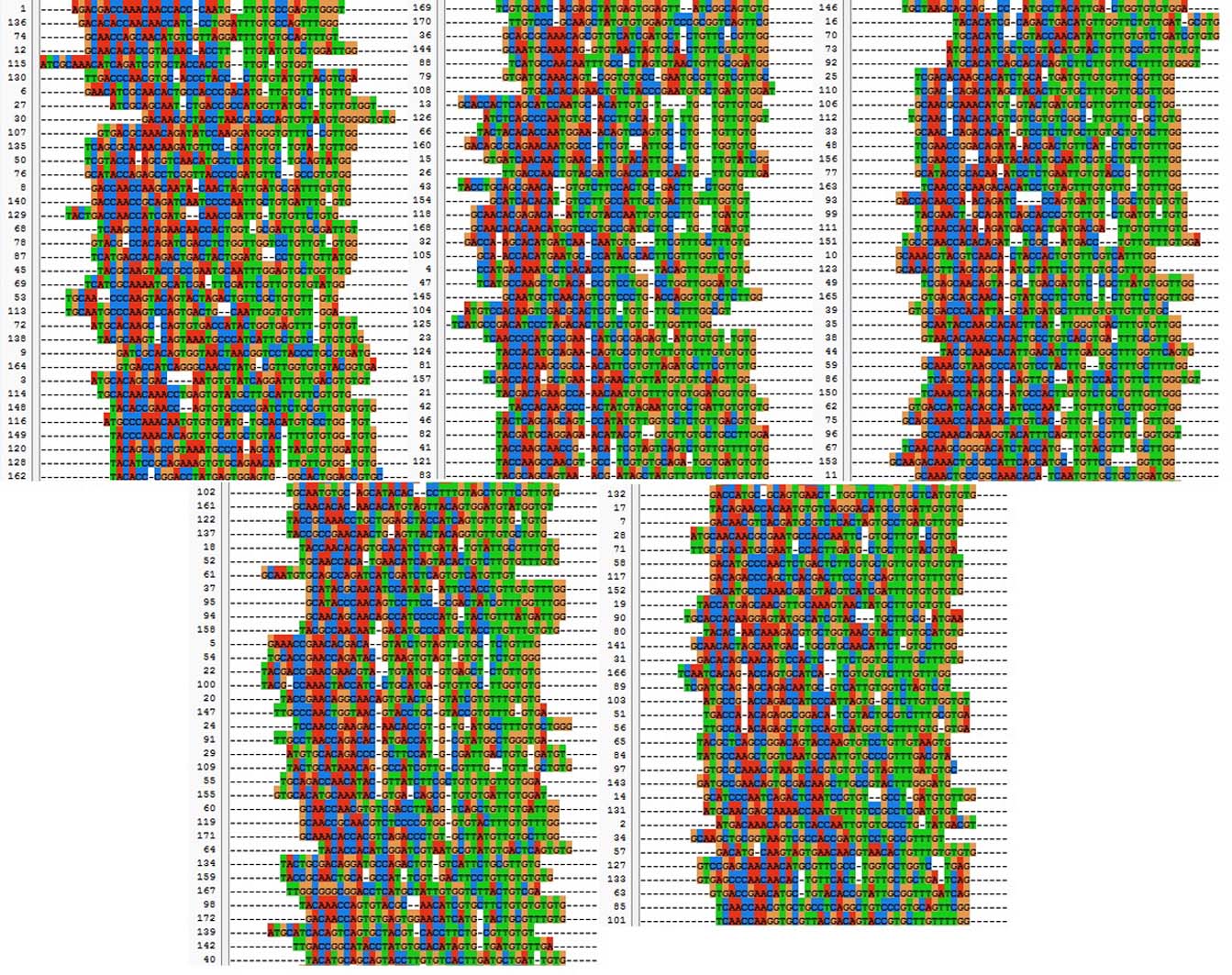

Supplement: Supplementary file 1 [file molecules-23-02337-s001.zip › Figure SI.jpg]

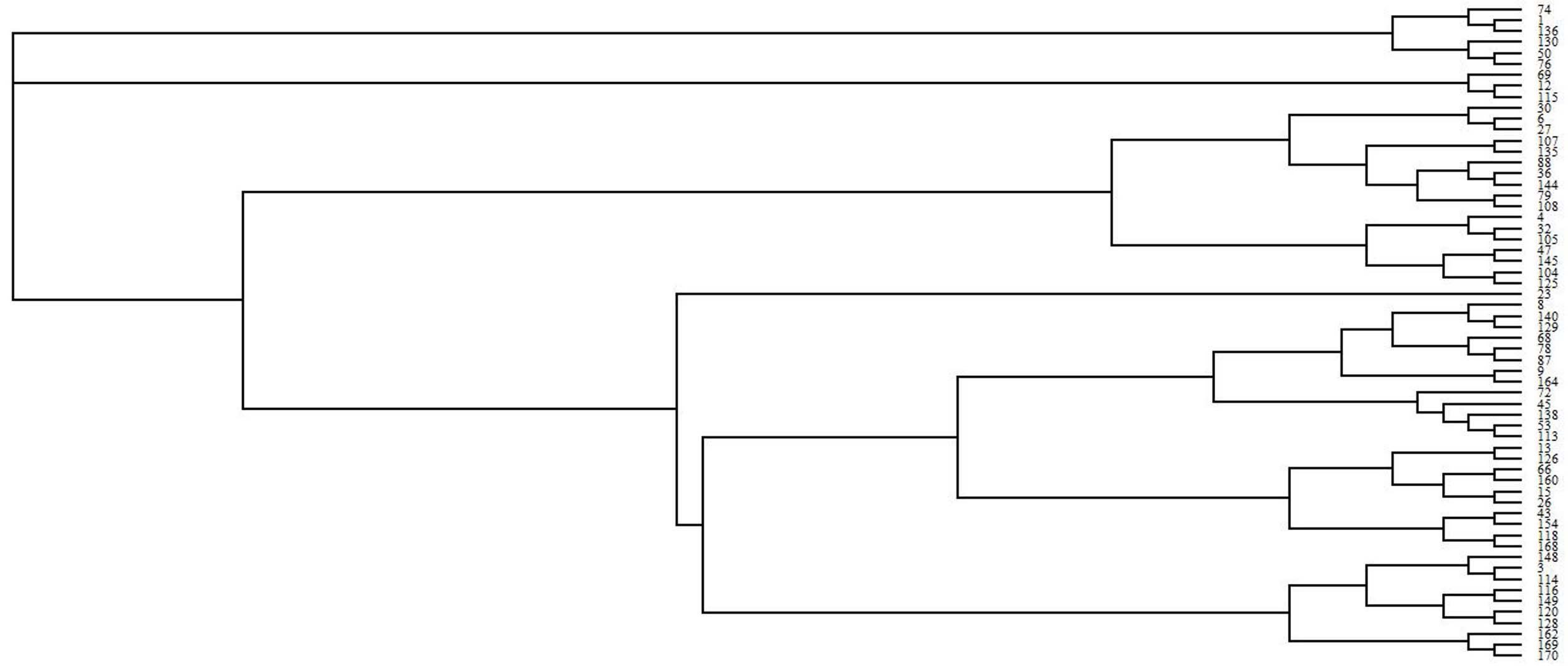

Supplement: Supplementary file 1 [file molecules-23-02337-s001.zip › Figure S2A.jpg]
